# Suitability Analysis of Machine Learning Algorithms for Crack Growth Prediction Based on Dynamic Response Data

**DOI:** 10.3390/s23031074

**Published:** 2023-01-17

**Authors:** Intisar Omar, Muhammad Khan, Andrew Starr

**Affiliations:** School of Aerospace, Transport and Manufacturing, Cranfield University, Bedford MK43 0AL, UK

**Keywords:** machine learning, K nearest neighbor, support vector machine, ridge regression, artificial neural network, least absolute shrinkage and selection operator (LASSO) regression, suitable machine learning model

## Abstract

Machine learning has the potential to enhance damage detection and prediction in materials science. Machine learning also has the ability to produce highly reliable and accurate representations, which can improve the detection and prediction of damage compared to the traditional knowledge-based approaches. These approaches can be used for a wide range of applications, including material design; predicting material properties; identifying hidden relationships; and classifying microstructures, defects, and damage. However, researchers must carefully consider the appropriateness of various machine learning algorithms, based on the available data, material being studied, and desired knowledge outcomes. In addition, the interpretability of certain machine learning models can be a limitation in materials science, as it may be difficult to understand the reasoning behind predictions. This paper aims to make novel contributions to the field of material engineering by analyzing the compatibility of dynamic response data from various material structures with prominent machine learning approaches. The purpose of this is to help researchers choose models that are both effective and understandable, while also enhancing their understanding of the model’s predictions. To achieve this, this paper analyzed the requirements and characteristics of commonly used machine learning algorithms for crack propagation in materials. This analysis assisted the authors in selecting machine learning algorithms (K nearest neighbor, Ridge, and Lasso regression) to evaluate the dynamic response of aluminum and ABS materials, using experimental data from previous studies to train the models. The results showed that natural frequency was the most significant predictor for ABS material, while temperature, natural frequency, and amplitude were the most important predictors for aluminum. Crack location along samples had no significant impact on either material. Future work could involve applying the discussed techniques to a wider range of materials under dynamic loading conditions.

## 1. Introduction

Machine learning approaches are crucial for allowing material engineers and scientists to accelerate the development of new materials, techniques, and processes. One of the main goals of using these approaches in materials science is to quickly identify and quantify key features along the process–performance–property–structure chain. The selection of the most suitable machine learning approaches for predicting structural damage should be carefully considered, taking into account the type of data available, the material being studied, the desired knowledge outcomes, the spatial and temporal scales, and the computational costs. Machine learning techniques can be used to identify sensitive features for material characterization, optimize and design manufacturing methods or novel materials, enhance complex measurement devices, or predict complex relationships that cannot be easily modeled using traditional methods. Due to the wide range of variables involved, machine learning is well-suited to tasks such as fault detection, failure mechanism classification, and predicting fatigue crack propagation under complex loads for different materials. Several studies have employed machine learning-based techniques to detect cracks in structures made of different materials. In one study, multiple linear regression, artificial neural networks, and adaptive neural–fuzzy inference systems were used to examine the relationship between the feature domain and crack intensity [1]. Another study combined proper orthogonal decomposition and artificial neural networks to predict the intensity and damage location of stringer-to-floor beam connections in riveted steel railway bridges under unknown, non-stationary train loads [2]. A combination of a genetic algorithm, fast Fourier transform analysis, and K-nearest neighbor algorithm was used to assess the health status of a spherical carbon steel tank [3]. In other research, deep learning, artificial neural networks, and recursive partitioning were used to predict the rating of cracks in metal pavements, using explanatory parameters such as asphalt thickness, roadway functional class, truck factor, and daily traffic [4]. Machine learning approaches have also been employed to predict the crack propagation or fatigue life of structures [5] and used with stochastic load and finite element analysis simulations, with random forests, neural networks, and support vector machine models, to predict the remaining fatigue life of slender coastal bridges under the effect of complex traffic loads, waves, and wind [6]. Haar wavelet discrete transform, random forests, and artificial neural networks were used to predict the severity and position of a crack in a Bernoulli cantilever beam [7].

A wide range of machine learning methods have been applied and proposed for damage detection in various structures. Artificial neural networks were used as a regression model to detect the crack propagation and formation in bridges [8], and the performance of neural networks, support vector machines, Gaussian processes, and random forests was compared in predicting the fatigue life of slender coastal bridges [9]. Artificial neural networks and recursive partitioning were used to predict the rating of cracks in highway pavements [4], and dynamic mode DE composite (DMD) and support vector machines were used to identify and classify defects in cantilever beams [9]. A novel anomaly detection method based on one-class K-nearest neighbors and Mahalanobis squared distance was proposed for structural health monitoring under varying environmental conditions and validated using two benchmarks: the Z24 Bridge and a wooden bridge [10]. K-nearest neighbors were used to classify microdamage in piezoelectric ceramics using ultrasound signals [11]. The fatigue performance of additive manufacturing processed 300M-AerMet 100 steel was investigated using machine learning, numerical simulation, and experiments [12]. Fuzzy logic, wavelet analysis, and artificial neural networks were used to locate, identify, and optimize the crack area and deterioration in civil infrastructure, such as reinforced concrete and concrete [12]. Adaptive neuro-fuzzy, multilayer perceptron neural networks, support vector machines, and least mean squares regression were used to predict the flexural crack spacing in ultrafine grained AL 2014 alloy [13]. Table 1 lists additional studies that have used different machine learning methods.

- Much of the current research in the field has focused on accurately predicting the severity or presence of structural damage, without sufficient explanation of why or how the predictions were made. This may be due to the complexity of certain machine learning models, which can be effective at handling complex problems but may not be easily interpretable or understandable. In general, the more complicated a model is, the more effective it may be at solving complex issues, but the less able it is to explain how and why predictions are made. This paper aims to analyze the requirements and characteristics of the most commonly used machine learning algorithms in materials science, in order to help researchers choose models that are both effective and understandable. This analysis can assist researchers in enhancing their understanding of the model’s predictions. This paper aims to make novel contributions to the field of material engineering by analyzing the compatibility of dynamic response data from various material structures with prominent machine learning approaches. By exploring the characteristics of the data and the most used machine learning algorithms in the field.

The rest of this article is organized as follows. Section 2 discusses some factors that can help in choosing an appropriate machine learning approach. The analysis process is described in Section 3, and the analysis of selected machine learning algorithms is presented in Section 4. The results of applying the selected algorithms to two different material datasets are shown in Section 5. The paper’s findings are summarized in the final section

## 2. Machine Learning Approach Selection

**Figure 1:**
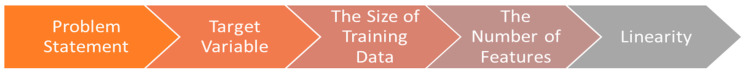
Some factors for selecting machine learning approaches.

In some structural health monitoring applications, the random selection of machine learning approaches [1,2,3], without clear justifications and a reliance on performance-based model selection, can lead to suboptimal results. To address this issue, this paper presents some factors (as shown in Figure 1) that can help in choosing the correct machine learning model for better performance and accuracy. Understanding the problem statement can determine whether it is a supervised learning problem (labeled dataset) or unsupervised learning problem (unlabeled dataset). The type of target variable (output of the model) can also narrow the search for a suitable machine learning approach. If the target variable is categorical, it is a classification problem. If the target variable is numerical, it is a regression problem. If the target variable is a set of input bunches, it is a clustering problem.

The size of the training data is an important factor to consider when selecting a machine learning approach, as it can impact the risk of underfitting and overfitting. If the dataset has a high number of features and a relatively small number of observations, it is advisable to choose machine learning approaches with low variance and high bias, such as linear SVM, linear regression, and naïve Bayes. On the other hand, if the number of observations is large compared to the number of features, approaches with high variance and low bias, such as decision trees, KNN, and kernel SVM, may be more suitable. The linearity of the data is also important to consider, as linear models such as linear regression, support vector machines, and logistic regression are well-suited for data that follows a straight-line trend. Non-linear models, such as neural networks, random forests, and kernel SVM, can handle complex and high-dimensional data. It is also important to consider the number of features, as a large number of features may slow down some learning algorithms and increase training time. In cases of data with a large number of features and relatively few observations, support vector machines are often recommended. Feature selection techniques and principal component analysis (PCA) can be used to select important features and reduce the dimensionality of the data, as discussed in [1].

The following subsections present an analysis some of the most commonly used machine learning techniques in structural health monitoring studies. This analysis can increase the probability of selecting suitable machine learning approaches and assist in interpreting and understanding the results of the model.

## 3. Analysis Process

**Figure 2:**
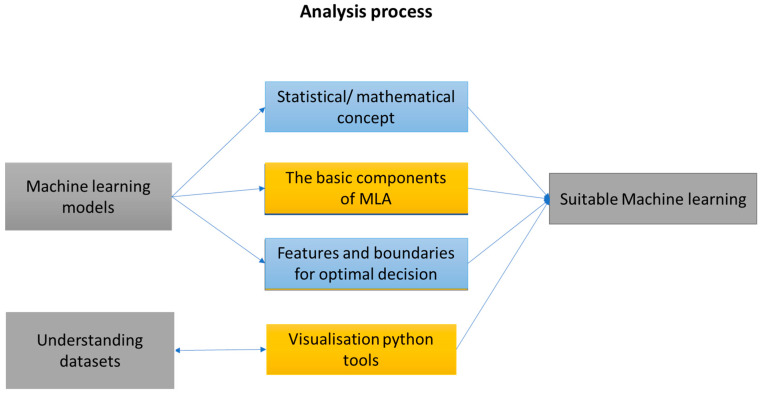
The analysis process of this paper.

This section presents the analysis process performed, as shown in Figure 2. The analysis aimed to understand the features and factors that impact the performance of machine learning techniques. This analysis can also provide useful guidance in choosing suitable machine learning techniques, which can help in understanding and interpreting the results of the model. Machine learning approaches can be selected based on the type of output in the used datasets (labels) or the size of the dataset (large or small). Therefore, the selected machine learning approaches (support vector machines, artificial neural networks, K-nearest neighbor, ridge regression, least absolute shrinkage, and selection operator (LASSO) regression) can be used to solve classification and regression problems. The analysis of commonly used machine learning approaches in structural health monitoring research is presented below:

### 3.1. Support Vector Machine (SVM)

Support vector machines (SVMs) are a type of supervised machine learning algorithm that can be used for both classification and regression tasks. They are known as kernel-based learning models because they use a kernel function to map the data from the input space to a higher-dimensional feature space, where it is easier to find an optimal separating hyperplane.

The goal of an SVM is to find the hyperplane that maximally separates the data points of different classes, while maximizing the margin. This ensures that the misclassification rate is minimized, and the model is robust to noise and outliers. SVMs have been successfully applied to a wide range of pattern recognition tasks and are known for their ability to handle high-dimensional data and class imbalances [15].

**Figure 3:**
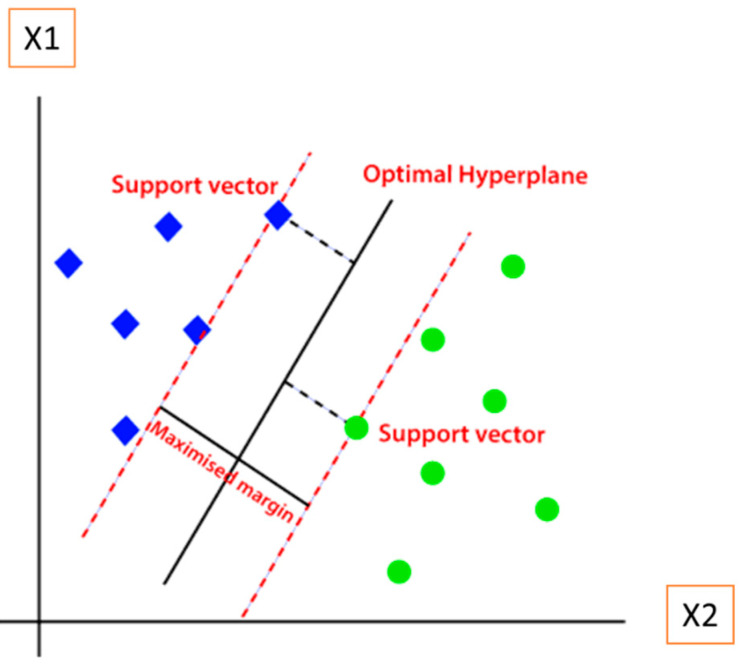
The components of SVM [17].

The basic components of an SVM include the support vectors, the optimal hyperplane, and the margin, as shown in Figure 3. The optimal hyperplane is the decision boundary that best separates the data points into different classes as green circles and blue squares in Figure 3. The dimensions of the hyperplane depend on the number of features in the dataset. For example, if the number of features is two, the hyperplane will be a straight line, and if the number of features is three, the hyperplane will be a 2-D plane. The support vectors are the data points that are closest to the hyperplane and have the greatest impact on its position. The margin is the space between the hyperplane and the support vectors [16,17].

The SVM can be applied to classify linear and non-linear datasets. If the input data are linearly separable and there are infinite hyperplanes that can perfectly separate the data. However, the generalization ability relies on the hyperplane with the maximum margin and the location of the separation hyperplane [18,19]. The mathematical formulation of a support vector machine model can determine the cost function. The boundary can be identified by

(1) $$0={\vec{w}}^{T}\cdot \vec{x}+b=\left(w_{1},w_{2}\right)\cdot \left(\begin{array}{c} x_{1}\\ x_{2} \end{array}\right)+b$$

Further two margin boundary equations are

(2) $$+\mathrm{margin}\;\mathrm{boundary}+1={\vec{w}}^{T}\cdot \vec{x}+b$$

(3) $$-\mathrm{margin}\;\mathrm{boundary}-1={\vec{w}}^{T}\cdot \vec{x}+b$$

The purpose of a cost function is to maximize the margin’s width, to find a separating boundary with a clear separation. This means data points of different classes are as far as possible away from the separating boundary (hyperplane) [20]. The cost function is derived in three steps. First, we will prove that the parameter vector $\vec{w}$ is perpendicular to the hyperplane. Let us select two arbitrary points of the separating boundary. The corresponding vectors are denoted by $\vec{g_{1}}$ and $\vec{g_{2}}$. The difference $\vec{g_{2}}-\vec{g_{1}}$ is a vector on the separating boundary. Thus, if the parameter $\vec{w}$ is perpendicular to the separating boundary, the dot product of $\vec{w}$ and $\vec{g_{2}}-\vec{g_{1}}$ is 0. As the data points *g*_1_ and *g*_2_ hold the equation for the boundary, when substitute *g*_1_ into Equation (1)

$$\Rightarrow \;{\vec{w}}^{T}\cdot \vec{g_{1}}+b=0\;with\;\vec{x}=\vec{g_{1}}$$

$$\Rightarrow \;{\vec{w}}^{T}\cdot \vec{g_{2}}+b=0\;with\;\vec{x}=\vec{g_{2}}$$

$$\Rightarrow \;{\vec{w}}^{T}\cdot (\vec{g_{2}}-\vec{g_{1}})+b=0$$

In the second step, the length of the parameter $\vec{w}$ needs to be small, in order to maximise the margin width (denoted by *d*). Maximizing the margin width allows finding the best separation with the largest distances of the data points to the separating boundary. To prove that this corresponds to a small length of $\vec{w}$, there has to be at least a single data point on each margin boundary. Otherwise, the space around the separating boundary without data could be larger. The vectors corresponding to these data points on the margin boundaries are the support vectors, these vectors support the determination of the model by limiting the margin width *d*. They give the algorithm its name of support vector machine [17,21]. Consider the data point vector $\vec{x_{1}}$ on the positive margin boundary and data point vector $x_{2}$ on the negative margin boundary, as follows:

$${\vec{w}}^{T}\cdot \vec{x_{1}}+b=1\;with\;\vec{x_{1}}+\mathrm{margin}$$

$${\vec{w}}^{T}\cdot \vec{x_{2}}+b=-1\;with\;\vec{x_{1}}-\mathrm{margin}$$

$$\Rightarrow \;{\vec{w}}^{T}\cdot \left(\vec{x_{1}}-\vec{x_{2}}\right)=1-(-1)$$

$$\Rightarrow \;{\vec{w}}^{T}\cdot \left(\vec{x_{1}}-\vec{x_{2}}\right)=2$$

Dividing the previous equation by the length of the vector $\left\|\vec{w}\right\|$, which is a unit vector and perpendicular to the boundary as

$$\Rightarrow \;\frac{{\vec{w}}^{T}}{\left\|\vec{w}\right\|}.\left(\vec{x_{1}}-\vec{x_{2}}\right)=\frac{2}{\left\|\vec{w}\right\|}$$

$$\Rightarrow \;d=\frac{{\vec{w}}^{T}}{\left\|\vec{w}\right\|}\cdot \left(\vec{x_{1}}-\vec{x_{2}}\right)$$

$$d=\frac{2}{\left\|\vec{w}\right\|}$$

Thus, we have an inverse relation between the margin width d and the length of the vector $\left\|\vec{w}\right\|$. This means small values for the length of $\left\|\vec{w}\right\|$ give large values for the margin width d.

Finally, the third step formulates the cost function and the corresponding constraints, in order to maximize the width of the margin, and it is necessary to minimize the length of $\left\|\vec{w}\right\|$. The square of the vector’s length $\left\|\vec{w}\right\|$ is used as cost function as

$$\frac{1}{d}=\frac{1}{2}{\left\|\vec{w}\right\|}^{2}\to min$$

Consequently, minimizing this cost function maximizes the margin width. However, without any constraints, this would give a vanishing vector length and thus an infinite large margin [17]. The constraint is that the margin needs to be free of data point inside, and the cost function with constraints is as below:

$$h\left(\vec{w},b\right)=\frac{1}{2}{\left\|\vec{w}\right\|}^{2}\to min$$

With constraints

$$y_{i}\left({\vec{w}}^{T}\cdot \vec{x_{i}}+b\right)\geq 1$$

With data encoding

$$y_{i}=\{\begin{array}{c} +1\;for\;class\;B\\ -1\;for\;class\;A \end{array}$$

The SVM operates the classifications through specifying some training datasets located in the support vectors, where the distance between them is augmented by an optimization function that reduces the hyperplane’s directional vector Euclidean norm. The SVM theory can be recommended for multiclass classification problems using strategies based on the binary classifier’s combination, such as one-against-all and one-against-one [22,23]. In case of non-linear problems, the input variables are represented in higher-dimensional feature space by utilizing a kernel function [19]. Table 2 demonstrates the most commonly used kernels and their characteristics. There is no consensus as to which kernel is worse or better for particular applications. Some scholars such as [24] have examined the performance of different SVM kernels, and their results confirmed that Gaussian RBF and polynomial were the best choices for damage detection using acoustic signals. On the other hand, [25] observed that Gaussian RBF and hyperbolic tangent were the best for genome-wide prediction. Less popular kernels might achieve better findings compared to the more common kernels. For instance [26] found that Laplace kernels were the best for intrusion detection. Scholars have confirmed that kernel selection should depend on the collected data characteristics, to achieve acceptable results. SVM has been shown to be a better choice than the other existing classification approaches. Although SVM has some drawbacks in terms of its algorithmic complexity, imbalanced datasets multi-class datasets, and parameter selection, SVM has been favored by many due to its good generalization performance and theoretical foundation. It is often employed for real-world classification problems. It is important to note that SVM is not very popular when datasets have a huge quantity of observations, because this requires a long training time [27].

### 3.2. K-Nearest Neighbor KNN

The KNN algorithm is a simple and effective method for both classification and regression tasks. It is a nonparametric algorithm, which means that it makes no assumptions about the underlying data distribution.

**Figure 4:**
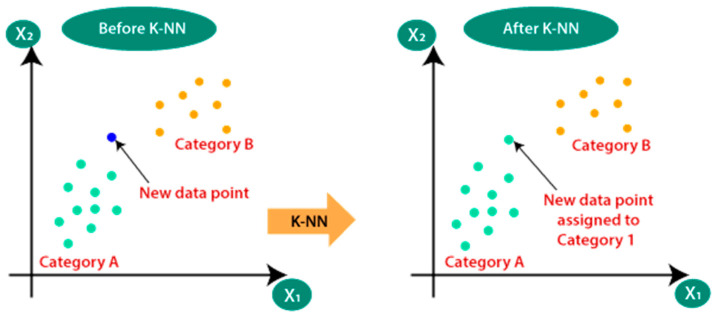
The idea of KNN classification [30].

In the KNN algorithm, the output (prediction) for a given data point is based on the values of its K nearest neighbors. The value of K is a hyperparameter that can be chosen by the user. For classification tasks, the output is typically the class label that is most common among the K nearest neighbors, as shown in Figure 4. For regression tasks, the output is the average of the values of the K nearest neighbors [28]. The KNN algorithm is relatively simple to implement and can be useful for tasks where the data is not linearly separable. It is also useful for tasks where the number of dimensions in the input space is high, as it does not rely on any explicit mapping of the data into a higher-dimensional space [28]. Overall, the KNN algorithm is a useful tool for many pattern recognition tasks and is often used as a baseline method for comparison with more complex algorithms [29].

The following points should be taken into consideration when selecting the ideal K value and calculating distance. There are no previous defined statistical approaches to locate the most satisfactory K value [30,31].

- Initialise a random K value and start calculation.
- Derive a plot between the error rate and K denoting values in a defined range. Then select the K value that decreases the error rate.
- Selecting a small value of K leads to unstable decision boundaries
- The significant K value leads to smoothening the decision boundaries.

The most commonly employed distance functions to compute the distance between the test data point and all training data points are Hamming distance (for categorical), Manhattan, and Euclidian (for continuous) [31].

- Hamming distance: This is applied for categorical variables (binary features). If the value (*x*) and the value (*y*) are not same, the distance *D* = 1. Otherwise, *D* = 0.
  $$\mathrm{Hamming}\ldots \ldots \ldots \ldots .\;D_{H}=\sum_{i=1}^{k}\left|x_{i}\right.-\left.y_{i}\right|$$
  with x=y⇒D=0
  x≠y⇒D=1
- Manhattan distance: The distance between points computed by the sum of their absolute difference.
  $$\mathrm{Manhattan}\ldots \ldots \ldots \ldots .\;\sum_{i=1}^{k}\left|x_{i}\right.-\left.y_{i}\right|$$
- Euclidean distance: This is computed by the square root of the sum of the squared differences between a new point (*x*) and an existing point (*y*).
  $$\mathrm{Euclidean}\ldots \ldots \ldots \ldots \;\sqrt{\sum_{i=1}^{k}{\left(x_{i}-y_{i}\right)}^{2}}$$

### 3.3. Ridge Regression and LASSO Regression

In linear regression, a linear relationship exists between the input features and the target variable. The association is a line in the case of a single input variable. However, the relationship between the input features and target variable can be considered as a hyperplane with higher dimensions. The optimization method can find the coefficients that minimize the error between the predicted output “$\hat{y}$” and the actual output “*y*” [32,33].

Overfitting/underfitting problems can cause unstable and inaccurate linear regression models. Therefore, the linear regression model includes a modified version of the loss function, referred to as “regularized or penalized linear regression” [34,35]. The regression model using the L2 regularization method is known as ridge regression. While the regression model applying L1 regularization is known as lasso “Least Absolute Shrinkage and Selection Operator” regression.

Ridge regression is a reasonable approach to reduce the collinearity between the regression predictors in the model. Collinearity occurs when one feature variable can be linearly predicted from the others with good accuracy in a multiple regression model [33], [36,37]. The L2 regularization aspect is represented by $\lambda \sum_{j=1}^{p}\beta_{j}^{2}$. The ridge regression is the squared magnitude of the coefficient “penalty” added to the loss function [35,38,39] as:

$$\sum_{i=1}^{n}{\left(y_{i}-\sum_{j=1}^{p}x_{ij}\beta_{j}\right)}^{2}+\lambda \sum_{j=1}^{p}\beta_{j}^{2}\;\ldots \ldots .\;\mathrm{Ridge}\;\mathrm{egression}$$

λ ≠ 0 (Lambda not equal 0)

Lasso regression is quite similar to ridge regression since both techniques have the same principle. Add a bias value to the regression optimization function to reduce the effect of model variance and collinearity [26,40,41]. However, lasso uses the absolute value of the bias, instead of using a square bias as in ridge regression.

$$\sum_{i=1}^{n}{\left(y_{i}-\sum_{j=1}^{p}x_{ij}\beta_{j}\right)}^{2}+\lambda \sum_{j=1}^{p}\left|\beta_{j}\right|\;\ldots .\;\mathrm{LASSO}\;\mathrm{Regresion}$$

In the formula above, ridge regression and lasso generate a set of coefficient estimates whose values depend on the various values of *λ*. The lambda should not equal zero. A high value of lamda will cause model underfitting, because it will add too much weight. Therefore, the parameter of lambda directly impacts on the accuracy of model predictions. Deciding a good value of *λ* is a significant step in both techniques. To achieve this task, a number of approaches have been outlined in the literature [36,37,39,42,43]. The most commonly applied method is k-fold cross-validation, where the data are divided into k subsets of approximately the same size and one of the subsets becomes the validation set. Residual k-subsets are used as training data. This process is repeated several times, each time with a different validation set, and the optimal value of *λ* is estimated, in order to maximize the cross-validated log-likelihood [43].

There are some variations between lasso regression and ridge regression [26,44,45]. This fundamentally affects the various properties of L1 and L2 regularization.

- Computational efficiency: The L1-norm solution has sparing properties that allow using it with sparse algorithms, making the calculation more efficient. However, L2 has an analytic explanation, but L1 does not. This enables the effective calculation of L2-norm solutions.
- The embedded “built-in” feature selection is commonly known as the effective property of the L1-norm. This is in fact the outcome of the L1-norm that generates sparse coefficients. The L2-norm generates non-sparse coefficients and, therefore, does not have this property. Thus, it may be said that lasso regression employs a form of “parameter selection”, since the feature variables that are not chosen will have a total weight of 0.
- Sparsity: belonging to the smallest number of entries in a matrix (vector) that are non-zero. L1-norm has the ability to generate very small values or zero values with few large coefficients. This is related to the earlier point that lasso can carry out a feature selection.

### 3.4. Artificial Neural Networks (ANN)

Artificial neural networks (ANNs) are a type of machine learning model inspired by the structure and function of the human brain. They are composed of interconnected neurons, or nodes, that are organized into layers. ANNs are used to solve a wide range of problems in various fields, including optimization and control, prediction, and pattern classification. There are several types of ANNs, including statistical ANNs, static ANNs, and dynamic ANNs. Statistical ANNs include models such as generalized regression neural networks and radial basis function models. Static ANNs, also known as multilayer perception neural networks, are the most commonly used type of ANN. Dynamic ANNs include models such as recurrent neural networks and tapped delay line models [46,47]. 

**Figure 5:**
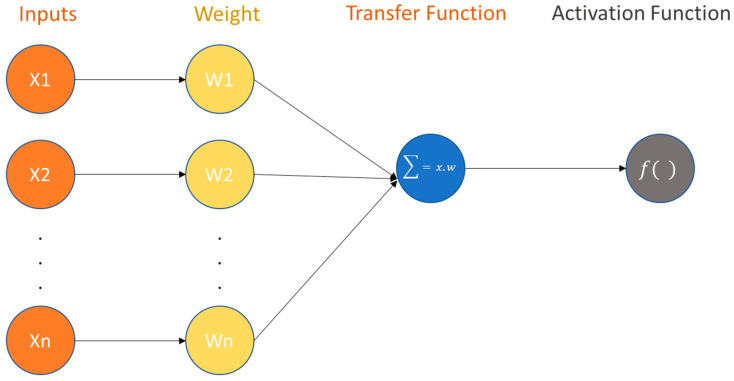
The structure of a neural network’s smallest component, the “perceptron” [47].

The basic component of an ANN is the perceptron, which is a single neuron that receives input features and produces an output. The perceptron consists of three primary mathematical operations: transformation by an activation function, scaler multiplication, and summation. These operations allow the perceptron to process and analyze the input features, and make a prediction based on the patterns and relationships in the data [47]. The structure of a perceptron is illustrated in Figure 5 and consists of three primary mathematical operations: transformation by an activation function, scaler multiplication, and summation. The activation function is a mathematical function that determines the output of the perceptron based on the input features. The activation function can be linear or nonlinear, depending on the task and the complexity of the data.

Scaler multiplication involves multiplying each input feature by a weight value, which represents the importance of that feature in determining the output. The weighted input values are then summed to produce the final output of the perceptron.

Overall, the perceptron uses these three mathematical operations to process and analyze the input features and make a prediction based on the patterns and relationships in the data.

Acquiring a strong understanding of the mathematics behind neural networks can be helpful in understanding how these models work and in performing tasks such as hyperparameter tuning, optimization, and architecture selection. To understand the mathematics of neural networks, it is useful to divide the processes involved into three main parts: forward propagation, back propagation, and optimization. 

**Figure 6:**
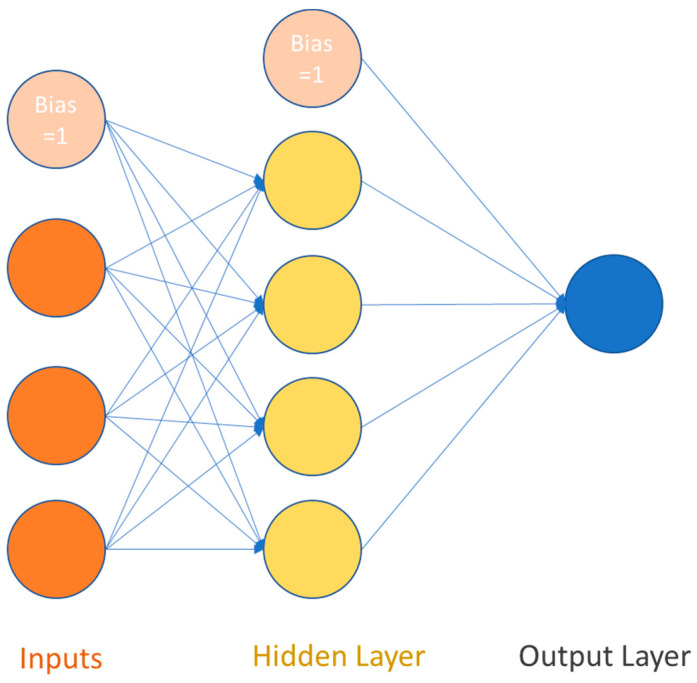
Forward propagation neural network [46].

Forward propagation (as shown in Figure 6) is the process of inputting a set of features into a neural network and using the mathematical operations of the perceptions to produce an output prediction. This involves transforming the input features through the activation function, multiplying them by the weights, and summing them to produce the output. This consists of the following three steps [46,48,49]:

**Step-1** Multiply each input value *x_i_* with weights *w_i_* and sum all the multiplied values. Weights (denoted as w or θ) are the strength of the connection between perceptrons and decide how much impact the given features will have on the prediction “output perceptron”. If the weight *w*_2_ has a higher value than the weight *w*_1_, then the feature *x*_2_ will have a higher impact on the model prediction than *w*_1_.

(4) $$\sum =\left(x_{1}\times w_{1}\right)+\left(x_{2}\times w_{2}\right)+\ldots +\left(x_{n}\times w_{n}\right)$$

The row vector of features and weights are $x=[x_{1},x_{2},\ldots ,x_{n}]$ and $w=[w_{1},w_{2},\ldots ,w_{n}]$ and their dot product is given by

(5) $$x\cdot w=\left(x_{1}\times w_{1}\right)+\left(x_{2}\times w_{2}\right)+\ldots +\left(x_{n}\times w_{n}\right)$$

Thus, the sum is equal to the dot product of the vectors *x* and *w*, also known as a transfer function. The purpose of the transfer function is to combine multiple inputs into one output value; thus, the activation function can be employed.

(6) $$\sum_{i=1}^{n}x_{n}\cdot w_{n}$$

**Step-2** Add bias b to the sum of the multiplied values denoted as z. Bias is also known as the offset. Bias is an additional input of 1. It is multiplied by a weight, the same as the other inputs. Bias is essential to permit the value before the activation function to be changed up and down, independently of the inputs themselves. This can balance the total sum to be around 0.

(7) z=x·w+b

**Figure 7:**
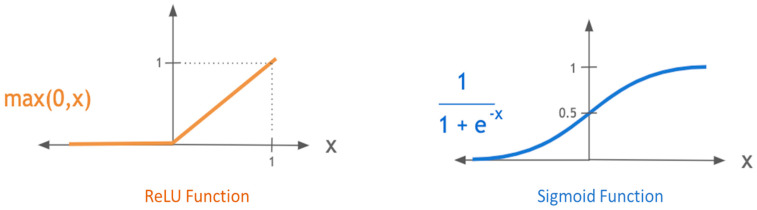
The common activation functions for neural networks [46,47,48].

**Step-3** Pass the z value to the one of non-linear activation functions. Activation functions (e.g., ReLU, Sigmoid, Tanh) are applied to introduce non-linearity into neuron’s output. This means that ANN without activation functions will only be a linear function. Furthermore, the activation function has an essential influence on the neural network learning speed. There are two commonly used activation functions: ReLU and Sigmoid, as shown in Figure 7.

The ReLU function compares zero with the inputs and selects the maximum. This means the positive inputs are unaffected, while any negative input becomes zero. The ReLU function is valuable where negative values do not make much sense or for eliminating linearity without heavy computation.

(8) $$\hat{y}=max\;(0,z)$$

The sigmoid function is effective at separating values into various thresholds. It is known as a logistic function and denoted as *σ*. This function ensures that the output of each neuron is always between 0 and 1.

(9) $$\hat{y}=\sigma \left(z\right)=\frac{1}{1+e^{-z}}$$

Neural network learning includes two processes: backward propagation and optimization. Backward propagation is the process of adjusting the weights and biases of the perceptrons in the neural network, in order to minimize the error between the predicted output and the true output. This is typically done using an optimization algorithm, such as stochastic gradient descent, which adjusts the weights and biases in a way that reduces the error. This is clarified in the following steps:

**Step-1** A loss function is applied to estimate how far the model prediction (predicted output) is from the actual output. Commonly, cross entropy is selected as the loss function for classification problems, with mean squared error for regression problems, as in Equation (10). The mean squared error squares the difference between the actual output (*y_i_*) and predicted output (${\hat{y}}_{i}$).

(10) $${MSE}_{i}={\left(y_{i}-{\hat{y}}_{i}\right)}^{2}$$

The loss function is computed for the entire training dataset and their average is called the cost function *J*.

(11) $$J=\frac{1}{n}\sum_{i=1}^{n}{\left(y_{i}-{\hat{y}}_{i}\right)}^{2}$$

**Step-2** It is essential to realize how the cost function changes to locate the best bias and weights for each neuron in the network. Thus, it should locate the gradient (change rate) of the cost function with respect to the bias (see Equation (12)) and weights (see Equation (13)).

(12) ∂J∂Wi=2n×sumy−y^×σz×1−σz

(13) ∂J∂Wi=2n×sumy−y^×σz×1−σz×xi

**Optimization**—This is the process of adjusting the weights and biases of the perceptions in the neural network in order to minimize the error between the predicted output and the true output. This can be done using various optimization algorithms, such as stochastic gradient descent, which adjusts the weights and biases in a way that reduces the error. To select the best weight and bias of the neuron, optimization algorithms such as gradient descent can be employed. The optimization algorithm adjustments the bias and weights related to the negative of the gradient of the cost function *J* with respect to the corresponding bias and weights (see Equations (14) and (15)) [50].

(14) $$b=b-\left(\alpha \times \frac{\partial J}{b}\right)$$

(15) $$w_{i}=w_{i}-\left(\alpha \times \frac{\partial J}{w_{i}}\right)$$

where α is the learning rate hyperparameter utilized to monitor how much the bias and weights are affected. It is important to take into consideration the following boundaries when identifying the shape of a neural network:

■ Determine the number of layers and number of neurons in each layer in the network.
■ The number of neurons in input layer should be equal to the number of features in the dataset.
■ In a regression problem case, the output layer should include one neuron.
■ With the increase in the number of hidden layers, the quality and prediction efficiency of the network increases, and the time required also increases.
■ Ref. [47] suggests that selecting the number of the hidden layer is dependent on the number of features in the dataset. In a dataset with fewer features, the neural network should contain no more than one to two hidden layers to reach an optimum prediction. Furthermore, no more than three to five hidden layers can be used for datasets with large dimensions, to avoid increasing the complexity of the model and overfitting problem.
■ The number of hidden neurons should be between the number of the target layer and features (input layer). Ref. [51] confirmed that the most appropriate number of hidden neurons can be calculated by $\sqrt{\left(input\;layer\;nodes*output\;layer\;nodes\right)}$.
■ Moreover, the following should be kept in mind when determining the initial values of Theta θ/w:
■ In linear regression, the initial values of θ can be zero but this is not acceptable in a neural network because this will make the values of θ equal in all layers and this will never change, which will make the network unable to work or change the values of θ.
■ The initial values of θ in a neural network should be small random values between (−0.001 and 0.001). Furthermore, initialisation methods such as He/Xavier can be used to determine the θ values. Overall, understanding the mathematics behind neural networks is important for understanding how these models work and for optimizing their performance.

In addition to the traditional machine learning techniques, deep learning techniques such as convolutional neural networks (CNNs) and recurrent neural networks (RNNs) have also been used for fatigue crack growth prediction [21,22,23,24,52]. These techniques have the ability to learn complex patterns and relationships in the data and have shown promising results in fatigue crack growth prediction tasks. It is worth noting that the choice of machine learning or deep learning technique will depend on the specific characteristics of the data and the goals of the analysis. Deep learning techniques can often achieve a high accuracy but may require a larger amount of data and more computational resources. At this point, advanced learning strategies (e.g., Bayesian optimization) and feature extraction approaches (e.g., PCA) can be applied to reduce the time needed for training and testing machine learning algorithms [16,25,26,47]. The use of two- or three-layer deep neural networks (DNNs) for fatigue crack growth rate prediction could potentially be effective, depending on the complexity of the problem and the amount of data available. One potential advantage of using DNNs for this task is that they are able to learn complex patterns and relationships in the data, which could be useful for accurately predicting crack growth rates. DNNs are also able to handle large amounts of data and can be trained on a wide range of tasks, making them a flexible choice for many machine learning problems.

However, there are also some potential limitations to consider when using DNNs for fatigue crack growth rate prediction. One potential issue is that DNNs can be prone to overfitting, especially when the amount of data available is limited. This can lead to a poor generalization performance on unseen data, which can reduce the reliability of the predictions made by the model. Another potential limitation is that DNNs can be resource-intensive to train and test, especially for larger and more complex models. This can make them less practical for tasks that need to be run in real-time or that have stringent computational constraints. In general, the use of DNNs for fatigue crack growth rate prediction could be a promising approach, but it is important to carefully consider the specific characteristics of the data and the requirements of the application before deciding whether this is the best model for the task. It may be helpful to experiment with different models and architectures to find the optimal configuration for your specific problem [27,29,40,41,44,45,53,54,55,56].

It is important to have a deep understanding of the raw data and the relationship between the independent and dependent variables, in order to select an appropriate machine learning model. Understanding the nature of the different machine learning models is also crucial in this process. There are various tools and techniques that can be used to help select an appropriate machine learning algorithm. Some techniques and tools are illustrated in the following sections. 

## 4. Data Description

**Figure 8:**
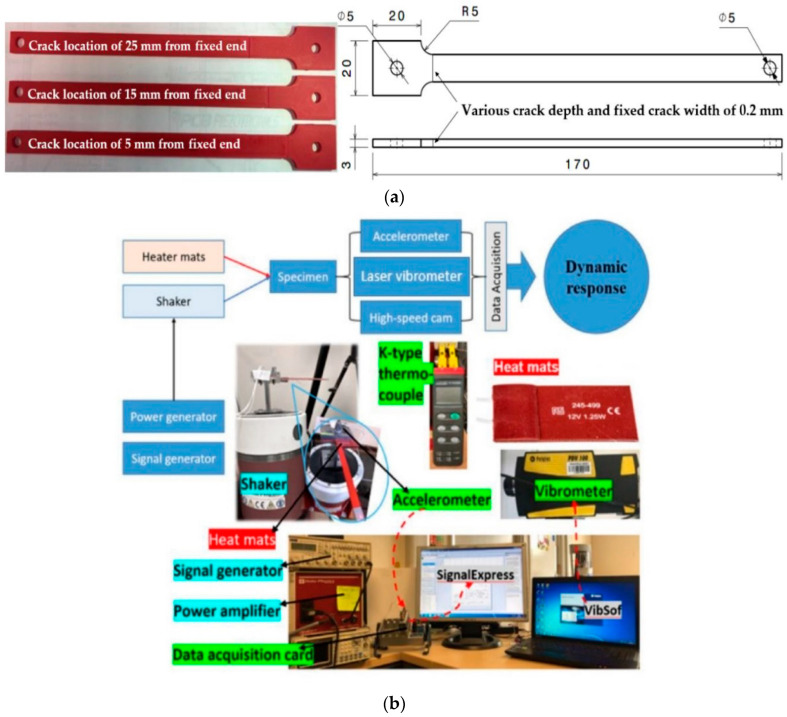
(**a**) Sample geometry, (**b**) the experimental setup.

Fused deposition modelling (FDM) 3D printed ABS and 2024 sheet aluminum were selected from previous studies [57,58] as representative materials. A variety of samples were manufactured using the geometry illustrated in Figure 8a. The gathered experimental data included the amplitude (mm), natural frequency (Hz), crack location (mm), temperature (C^0^), and crack depth (mm) for each observation. 

The experimental setup is illustrated in Figure 8b. The sample was fixed on a shaker at different temperatures. The mechanical loads were controlled by the shaker. An impact test was conducted to identify the fundamental frequency of the samples, and this was calculated using a laser vibrometer. In the case of crack propagation, the amplitude continued to be reduced. Therefore, the shaker was stopped to measure the new frequency. The impact test was conducted again to identify the new basic frequency. The new essential frequency was maintained until the next amplitude drop. This process refers to the testing of fatigue crack growth rate in a sample. This process was repeated until the sample experienced catastrophic failure, due to the propagation of cracks. More details about the experimental setup and methods can be found in references [27,53].

**Figure 9:**
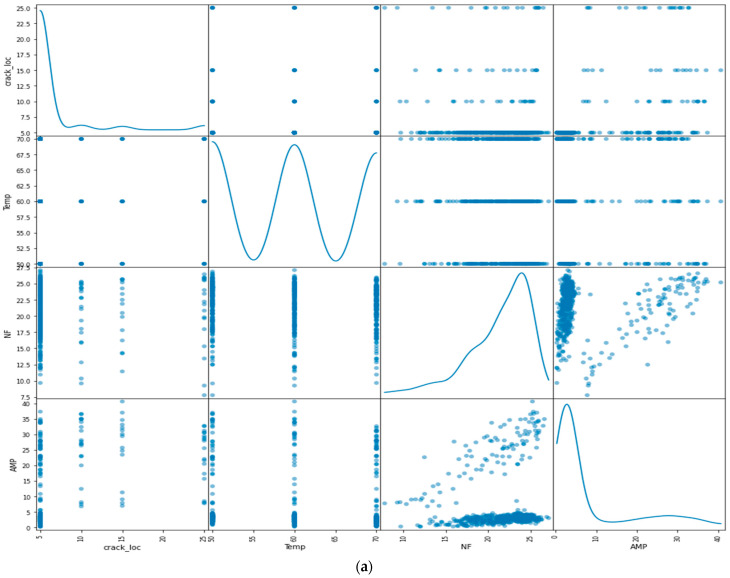

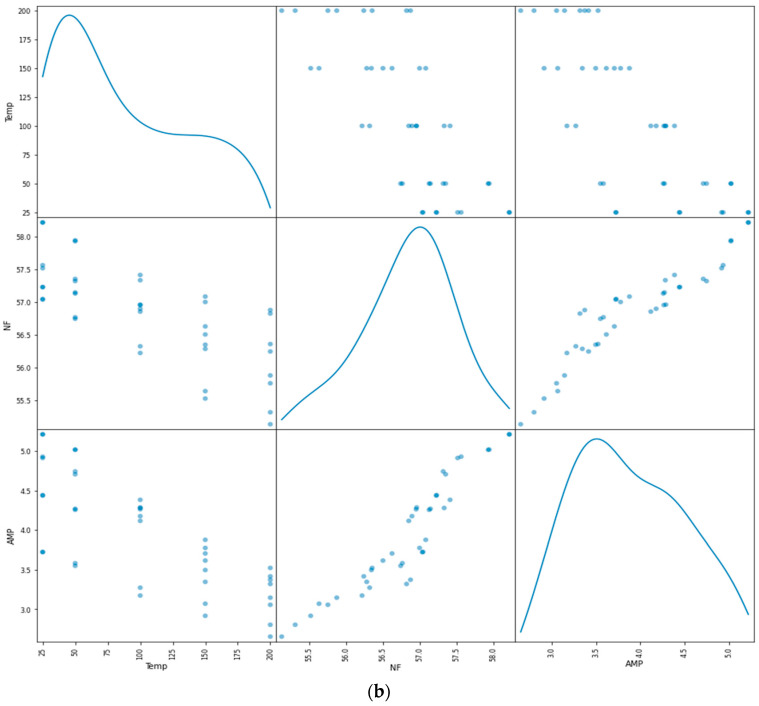
Scatter matrix for the relationship between the feature and the target variables of (**a**) polymer data (**b**) aluminum 2024-T3 data.

Jupyter Notebook was used to create and share documents that contained live code, equations, visualizations, and narrative text. It was used for data analysis, machine learning, and scientific computing, and it is a popular choice among practitioners and researchers in these fields. Before using a dataset for machine learning, it is often necessary to perform data analysis and feature extraction, in order to select the most relevant and informative features for the task at hand. This can be done using various techniques, such as visualizing the correlations between the features and the target. Visualization libraries, such as scatter matrix in Pandas, and the pair plot and grid plot in Seaborn [59,60,61] can be useful tools for understanding data and selecting a suitable machine learning model. These plots can help to visualize the relationships between the features in a dataset and can be used as part of a global sensitivity analysis (GSA), to evaluate the importance of the features for the output variables. GSA is a method of evaluating the sensitivity of the output of a model to changes in the input variables. It allows the entire proposed variable space to be considered, in order to discover real correlations between the feature and target variables. By integrating GSA with machine learning approaches, it is possible to gain a better understanding of the “black box” nature of many machine learning models and to make more informed decisions about which model to use for a given problem [62,63]. However, it is important to keep in mind that visualizations and GSA methods should be used in conjunction with an understanding of the nature of the different machine learning models and how they handle different types of problems. This can help to ensure that the appropriate model is selected for a given problem. The experimental data consisted of four features: crack location, temperature, natural frequency, and amplitude, and a predicted value: crack depth. The data were plotted using a scatter matrix, pair plot, and grid plot, to investigate the relationship between the features and the predicted value. Figure 9 illustrates a scatter matrix plot for both materials polymer and aluminum 2024-T3 data. The plots revealed that the data overlapped and that the target values were numerical, indicating that the task was a regression problem. The size of the dataset was 560 observations.

Before splitting the data, it is often useful to preprocess it using libraries such as Pandas and Numpy. These libraries provide tools for cleaning and formatting the data, as well as for performing basic statistical analysis. Using these techniques, you can prepare and preprocess the data in a way that allows for effective training and evaluation of the selected machine learning models. VarianceThreshold is a feature selection method used to drop features that have a constant or near-constant value. These types of features are often called “constant features,” and they generally do not provide any useful information for training a machine learning model. Removing them can help to reduce the complexity of the model and improve its performance. It is also important to consider scaling the continuous features in your dataset, especially if they have different units or ranges. Scaling the data can help to improve the training time of the model, as well as to ensure that the features are at a comparable scale. This can be done using a library such as StandardScaler, which performs standardization by subtracting the mean and dividing by the standard deviation of each feature. Once the data have been cleaned and prepared, they are typically split into a training set (80%) and a testing set (20%). The training set is used to train the model, while the testing set is used to evaluate the performance of the model on unseen data. This helps to ensure that the model is not overfit to the training data and can generalize well to new, unseen data. By using these techniques, it is possible to prepare and split the data in a way that allows for effective training and evaluation of the selected machine learning models.

Suitable machine learning models for this dataset may include K-nearest neighbors (KNN), ridge regression, and lasso regression. These algorithms were chosen based on the following factors:

- KNN is a non-parametric method that can be used for both classification and regression tasks. It is simple to implement and can be effective for small datasets.
- Ridge regression: Ridge regression is a linear model that is used for regression tasks. It is particularly useful for dealing with multicollinearity, which can occur when there are strong correlations between the features in the dataset.
- Lasso regression: Similarly to ridge regression, lasso regression is a linear model that is used for regression tasks. It is known for its ability to perform feature selection by setting the coefficients of unimportant features to zero. This can be useful for reducing the complexity of a model and improving interpretability. It is suitable for small datasets and prediction problems.
- These models do not require a long time or a lot of computational power for training and testing. This makes them a good choice for tasks that need to be run quickly or on resource-constrained systems.
- They are relatively simple and easy to understand, making them a good choice for those who are new to machine learning or do not have a strong background in mathematics. This can make it easier to implement and debug the models, as well as to interpret the results.
- They are easy to code and implement using the Scikit-learn library, which is a popular machine learning library in Python. This can make it faster and easier to get started with these algorithms, as well as to integrate them into larger systems.

Overall, these algorithms offer a good balance of performance and simplicity, making them a desirable choice for this dataset. It is always important to evaluate the performance of different models, in order to determine the most suitable one for a given task. The next section presents the results of applying the selected machine learning techniques on the prepared data. This includes an evaluation of the performance of the different models and an analysis of the results obtained. It is important to carefully examine the results, in order to determine which model performs the best in the given task and to identify any potential areas for improvement.

## 5. Results and Discussion

The main purpose of this research was not to generate an accurate prediction model, but to employ machine learning models to shed light on the theory, and an interpretable model is a fundamental aspect. Therefore, the selected machine learning models (KNN, ridge regression, and lasso) were investigated. As a result of the different material behaviors, and to permit sufficient comparison of the coefficients, two homogeneous but independent prediction models were trained: one for fused deposition modelling (FDM) 3D printed ABS and one for 2024 sheet aluminum. The proposed algorithms were trained using two separate datasets. As a result, it was not necessary to classify the model results based on the type of material. The algorithms could be trained on each dataset individually and then used to make predictions for fatigue crack growth rates for that specific material. This approach allowed for more accurate and reliable predictions, as the models could be specifically tailored to the properties and characteristics of each material. However, several studies have classified the results of a prediction model based on blind data, as indicated in [64].

**Figure 10:**
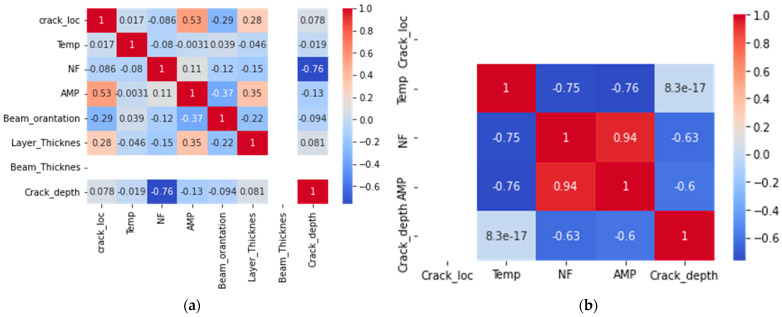
The heatmap indicates the effect of the features on each other in (**a**) ABS, (**b**) aluminum.

In this research, the experimental data consisted of measurements of the structural amplitude and natural frequency of aluminum and ABS specimens. It was found that the natural frequency of both materials showed similar patterns, with the frequency decreasing regularly with increasing crack depth and temperature. However, the effect of temperature on amplitude and natural frequency was more pronounced in the aluminum specimens than in the ABS specimens. This is likely due to the relative consistency and high elastic modulus of the isotropic properties of the aluminum plate compared to the additive layer in the ABS specimens. For the aluminum specimens, increasing the temperature and crack depth resulted in increasing the amplitude. This was expected, due to the damaging effect of a crack on the sample, which leads to a decrease in the natural frequency. However, the ABS specimens showed the opposite trend, with the natural frequency decreasing with increasing crack depth as in the aluminum specimens, but the amplitude decreasing rather than increasing. This may be due to the fact that the maximum temperature tested in the ABS specimens (70 °C) was close to the glass transition temperature, while the maximum temperature for the aluminum specimens (200 °C) was much lower and did not result in any significant material transformations. Figure 10 provides a more detailed view of the relationships between the features in the experimental data.

**Figure 11:**
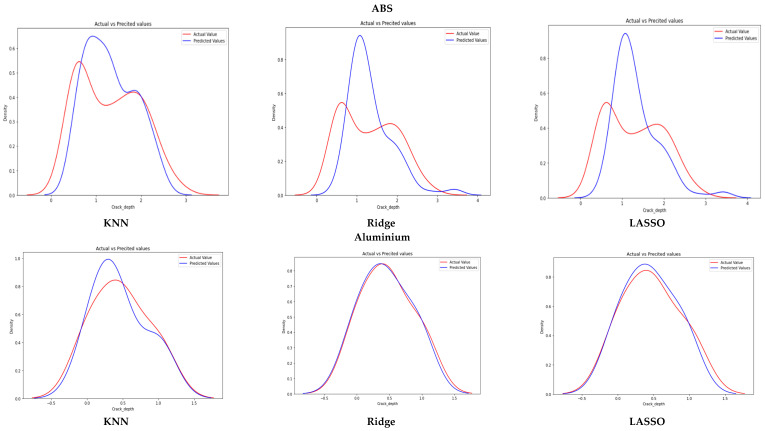
The model prediction values plotted against the actual crack depth measurements for ABS and aluminum.

The prediction accuracy of the selected machine learning models was measured using the root mean squared error (RMSE) for both materials. In general, a lower RMSE value indicates a better performing model, as it indicates a smaller average prediction error. Figure 11 shows the model prediction values of KNN, ridge, and lasso regression plotted against the actual crack depth measurements for both materials. Table 3 summarizes the performance metrics for the different models. The ABS model had a slightly worse performance than the aluminum model. Due to less connections between a propagating crack and the material layers, the manufactured ABS additive layer had a less isotropic nature and higher variance. This corresponds to the lower degree of variance of the model compared to the aluminum data [65].

Temperature, natural frequency, and amplitude seemed to be the most essential predictors for aluminum, but ABS seemed to be controlled by natural frequency. Interestingly, the crack location along the sample seemed to have little importance for both materials. This is illogical, as theory indicates that the closer the crack is to a specific location on the sample, the more obvious its influence on the dynamic response. The elimination of crack location and amplitude did not substantially raise the error. The temperature elimination had more effect on the error, increasing by approx. 40% relative to all other features.

Removing natural frequencies from selected features had a greater effect on the result accuracy. The results suggest that the natural frequency was the dominant feature in predicting crack depth and had a greater impact on model error than the rest of the features combined. The KNN models performed better, with a high accuracy, on ABS compared to the ridge and lasso regressions. Ridge and KNN handled the aluminum data with 96% accuracy.

The models utilized in this paper, including KNN, ridge regression, and lasso regression, are generally considered to be white-box models [66], meaning that they are relatively easy to interpret and understand. These models make their predictions based on a set of weights or coefficients that are applied to the input features and can be examined, to understand how the model is making its decisions. This makes it possible to understand the importance of each feature and how it contributes to the overall prediction, as well as to identify any potential biases in the model. However, it is important to note that even white-box models can be difficult to fully interpret, especially when working with large or complex datasets. It may also be challenging to fully understand the reasoning behind the model’s predictions when working with more complex models, such as deep neural networks. In these cases, it may be necessary to rely on other methods, such as feature importance analysis or model agnostic techniques such as LIME [67,68,69], to gain a better understanding of the model’s decision-making process. 

The computational time and complexity of training machine learning algorithms can vary significantly, depending on a number of factors, including the size and complexity of the dataset, the type of algorithm being used, and the hardware used to train the model. In general, however, the algorithms used in this paper (KNN, ridge regression, and lasso regression) are relatively fast and efficient, especially when working with small datasets such as the one described in this paper.

KNN is a relatively simple and fast algorithm that requires minimal training time, as it does not need to learn any model parameters. It simply stores the training data and makes predictions based on the closest neighbors to a given test point. Ridge regression and lasso regression are also relatively fast, especially when working with small datasets, as they only need to learn a small number of model parameters. Both algorithms typically scale well with increasing dataset size, although they may become slower when working with very large datasets or when using advanced optimization techniques. Overall, it is likely that the training time for the algorithms proposed in the paper would be relatively fast, even when working with small datasets. However, it is always a good idea to carefully consider the computational requirements of any machine learning model and to select the most appropriate algorithm, based on the available resources and requirements of the problem at hand.

Using ridge, lasso, and K-nearest neighbors (KNN) for crack prediction in small datasets with four features can be a challenging task, due to the limited amount of data available. In this case, it is important to carefully consider the strengths and limitations of each method, in order to choose the most appropriate one for the given task.

Ridge and lasso are both types of regularized linear regression, which means that they are designed to fit linear models to data, while imposing a penalty on the size of the model parameters. This can be helpful in preventing overfitting, which is a common issue when working with small datasets. However, these methods may not be well-suited to predicting the presence or location of cracks, as they are primarily designed for predicting continuous variables.

KNN, on the other hand, is a non-parametric method that can be used for both classification and regression tasks. It works by finding the K-nearest neighbors of a given data point and using the labels or values of these neighbors to make a prediction. This approach can be effective for small datasets, as it does not require the specification of a functional form for the relationship between the predictors and the response. However, KNN can be sensitive to the choice of K and may not perform well if the classes in the data are highly imbalanced.

In summary, while all three methods may have some potential for use in crack prediction with small datasets, they each have their own limitations and may not be the best choice in all cases. It may be useful to try multiple approaches and compare their performance, in order to determine the most effective method for the given data.

## 6. Conclusions

Machine learning techniques have the potential to significantly improve the detection and prediction of damage in materials science. However, it is important for researchers to carefully consider the suitability of different machine learning algorithms for their specific needs, taking into account the type of data available, the material being studied, and the desired knowledge outcomes. In addition to the points already mentioned, it is worth noting that the interpretability of certain machine learning models can be a limitation in the field of materials science. While these models may be able to produce highly accurate predictions, it may be difficult to understand the reasoning behind the predictions or to explain them to others. This lack of interpretability can be a drawback for researchers who want to understand the underlying relationships and mechanisms at play. Therefore, it is important for researchers to consider not only the predictive accuracy of a model, but also its interpretability when selecting a machine learning algorithm for their needs. The authors of this paper analyzed several machine learning algorithms commonly used in materials engineering and applied them to evaluate the dynamic response of aluminum and ABS materials. The results showed that natural frequency was the most significant predictor for the ABS material, while temperature, natural frequency, and amplitude were the most important predictors for aluminum. The location of cracks along the samples was found to have no significant impact on either material. The KNN, ridge, and lasso algorithms are suitable choices for machine learning tasks that need to be run efficiently and do not require a lot of computational power for training and testing. This is because these algorithms are relatively simple and do not have a large number of hyperparameters to tune, which can make them faster to train and test than some other models. These algorithms are also relatively easy to understand and interpret, which makes them a good choice for practitioners who are new to machine learning or do not have a strong background in mathematics. They are easy to code and implement using popular machine learning libraries such as Scikit-learn, which is widely used in the Python ecosystem. These algorithms are good options to consider when building machine learning models for tasks that need to be run efficiently and are relatively simple in nature. It is worth noting that these algorithms may not be the best choice for all problems, and it may be necessary to consider other models, depending on the specific characteristics of the data and the requirements of the application.

There are several potential directions for future work based on the conclusion of this paper. One potential avenue for future research would be to apply the machine learning techniques discussed in this paper to a wider range of materials under dynamic loading conditions. This could help to further validate the findings of the current study and to determine the generalizability of the results. Overall, the use of machine learning techniques in materials science has the potential to greatly improve our understanding of material behavior and to facilitate the development of new materials and processes. By carefully selecting the most suitable machine learning algorithms and properly evaluating their performance, researchers can make the most of this powerful tool.

## Figures and Tables

**Table 1:** More studies employing different machine learning methods.

Ref.	Damage Type	Structure	Extracted Features	MLA
[1]	Simulated stiffness reduction	FE model of a frame structure	Mode shapes and natural frequencies	Multilayer feedforward ANN
[2]	Simulated stiffness reduction	FE model of an RC slab	Natural frequencies and mode shapes	Two stage ANN
[3]	Elements removal from the FE model	FE model of a steel frame	Interval modeling	Adaptive neuro-fuzzy inference system (ANFIS)
[4]	Simulated stiffness reduction Free vibration	Analytical truss model	Mode shapes and natural frequencies	Multilayer feedforward ANN
[5]	Simulated stiffness reduction Free vibration	Analytical beam and frame models	Natural frequencies and mode shapes	Multi-stage ANN
[6]	Grinding slots in the flange	Simple supported I-beam	Mode shapes	Ensemble of ANNs
[7]	Saw cuts in the columns	Three-story frame structure	Natural frequencies and mode shapes	Multilayer feedforward ANN
[8]	Simulated stiffness reduction	7-DOF analytical model	Modal parameters discretized by K-means clustering	Probabilistic neural network
[9]	Saw cuts	Plate, beam, and shell	The mode shapes wavelet transform	Multilayer feedforward ANN
[10]	Schematic of the active damage detection	Laboratory-scale wind turbine with hollow composite blades	A set of statistical features	Logistic regression and support vector machine
[11]	Finding whether there is any relation between reduction in local stiffness and disruption of wavelet responsw coefficients because of the local bending stiffness reduction.	Steel gas pipelines	Frequencies and Amplitude	Support vector machine
[12]	Identify damage types and severity based on mode converted wave strength.	FE model of Composite laminated beam	Standard deviation, maximum amplitude, the energy of the segment:	Support vector machine and PCA
[13]	Detect crack depth and crack	Cantilever beam	Three natural frequencies	LASSO and Ridge regression models
[14]	ML prediction of stiffness degradation of the laminates	A small amount of finite element analysis of composite laminates	In-plane elastic properties of the composite lamina (E1, E2, v12, and G12), thickness of 90° ply (t2),crack density of 90° ply (ρ), and thickness of 0° ply (t1)	LASSO model

**Table 2:** The most commonly used SVM kernels and their characteristics.

Kernel Name	Parameters	Expression	Characteristics
Linear	None	$k\left(x_{i},x_{j}\right)=\langle x_{i},x_{j}\rangle +1={x_{i}}^{T}x_{j}+1$	SVM non-kernelised version is the simplest kernel function. Datasets with numerous features frequently become linearly separable problems. Therefore, this kernel is a good choice in these instances.
Gaussian radial basis function (RBF)	σ	$k\left(x_{i},x_{j}\right)=\exp\frac{{\left\|x_{i}-x_{j}\right\|}^{2}}{2\sigma^{2}}$	The most commonly employed in current studies, it can be used as a general purpose transform invariant kernel. Other related functions include the Laplace kernel and the exponential kernel. Parameter σ must be carefully selected.
Hyperbolic tangent	β,b	$k\left(x_{i},x_{j}\right)=tanh\;\langle x_{i},x_{j}\rangle >\beta +b$	This kernel is recognized as a sigmoid kernel, it is also utilized as an activation function in neural networks. b can be seen as a shift, and β as a scaling parameter of the product ${x_{i}}^{T}x_{j}$. These parameters impact significantly on the SVM performance.
Polynomial	$r\in Z^{+}$	$k\left(x_{i},x_{j}\right)={\left(\langle x_{i},\left.x_{j}\right)\rangle +1\right)}^{r}$	Maps the input space into a high-dimensional space that is a combination of polynomials products. Despite being computationally expensive, this kernel is often applied to normalized data

**Table 3:** The performance metrics for selected machine learning algorithms.

*ML Model*	Metrics	ABS	Aluminium	Accuracy Score	
				ABS	Aluminium
*KNN*	RMSE	0.39	0.08	0.67	0.96
*Ridge regression*	RMSE	0.18	0.006	0.62	0.96
*Lasso regression*	RMSE	0.17	0.064	0.62	0.95
